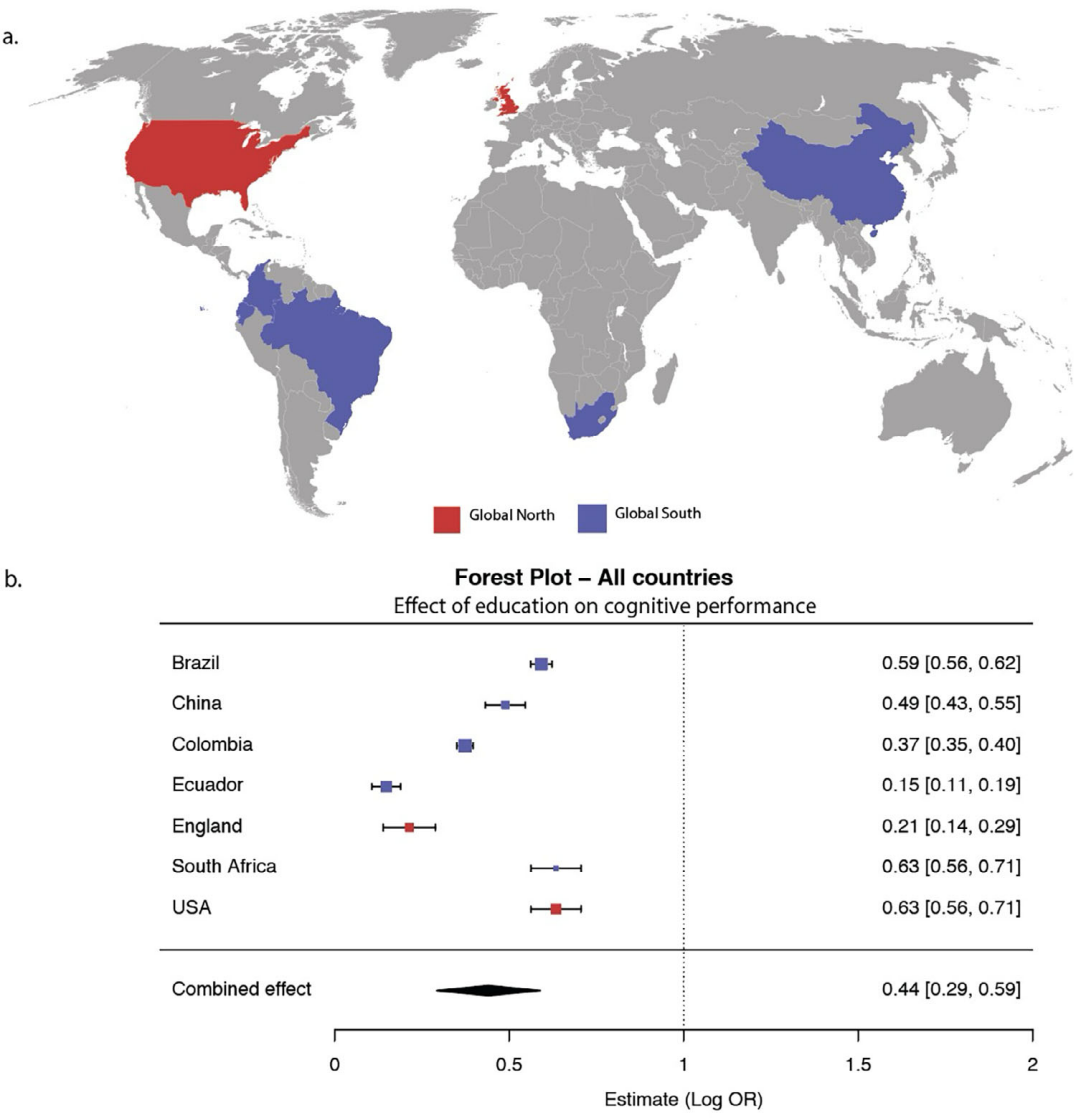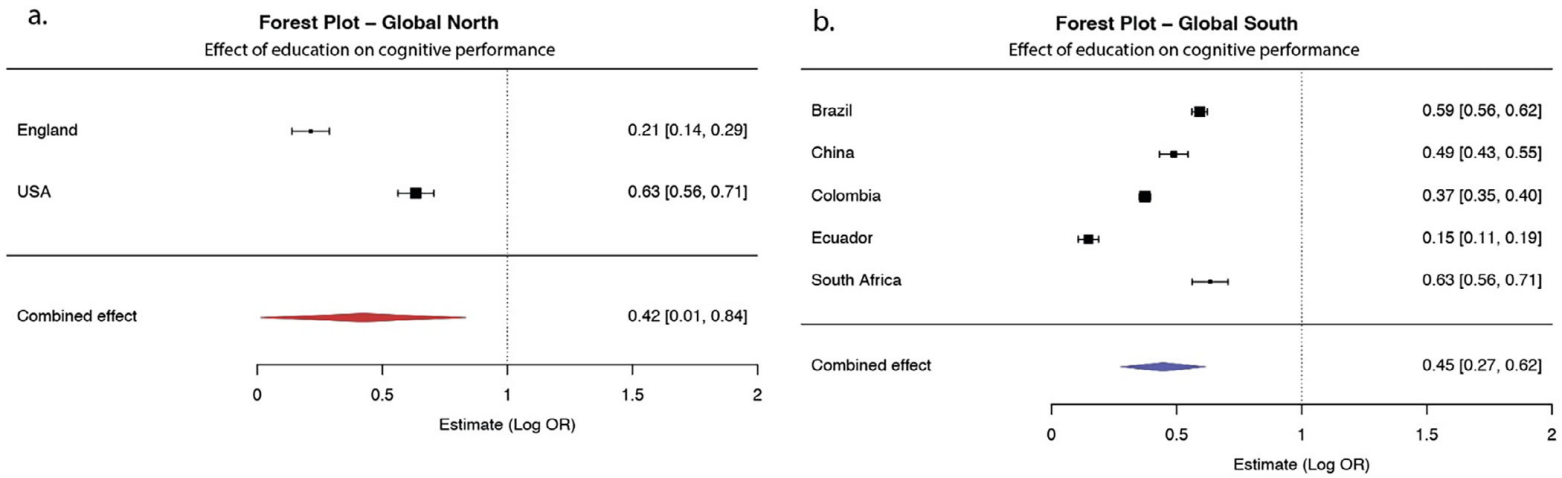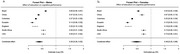# Impact of education on cognitive decline: meta‐analysis of representative population surveys from 4 continents

**DOI:** 10.1002/alz70857_105678

**Published:** 2025-12-25

**Authors:** João Pedro Uglione Da Ros, Lucas Uglione Da Ros, João Pedro Ferrari‐Souza, Marco De Bastiani, Joana Emilia Senger, Wyllians Vendramini Borelli, Eduardo R. Zimmer

**Affiliations:** ^1^ Universidade Luterana do Brazil, Canoas, RS, Brazil; ^2^ Universidade Federal Do Rio Grade Do Sul, Porto Alegre, Rio Grande do Sul, Brazil; ^3^ Universidade Federal do Rio Grande do Sul, Porto Alegre, RS, Brazil; ^4^ Universidade Federal do Rio Grande do Sul, Porto Alegre, Rio Grande do Sul, Brazil; ^5^ Centro de Memória, Hospital Moinhos de Vento, Porto Alegre, RS, Brazil; ^6^ Brain Institute of Rio Grande do Sul (InsCer), PUCRS, Porto Alegre, Rio Grande do Sul, Brazil

## Abstract

**Background:**

Social and health disparity factors have recently been shown to impact brain health more than demographic factors in Latin American countries. Data from Brazil suggests that education is the most significant contributor to cognitive decline. However, it remains to be elucidated whether the neuroprotective effects of education are restricted to specific regions across the world. This study aims to provide a global estimate of the influence of education on cognition, stratifying by global north and south.

**Method:**

We included data from epidemiological datasets from Brazil (*n* = 9,412), China (*n* = 17,920), Colombia (*n* = 23,694), Ecuador (*n* = 5,235), England (*n* = 1,273), South Africa (*n* = 5,059), and the U.S.A. (*n* = 42,406). All datasets were designed to evaluate health conditions, income, work, health insurance, disability, physical health and functioning, and cognitive functioning in elderly individuals from representative samples of their respective populations. Each individual was categorized into one of three education levels: up to elementary school, up to high school, and higher degrees. A random‐effects meta‐analysis was then conducted to estimate combined predictor effects. Effect estimates and sample‐size‐adjusted standard errors were included, with predictors as moderators using the metafor package in R.

**Result:**

We included 104,999 individuals from four continents. The combined estimate from our meta‐analysis was 0.44 (*p* < 0.0001), demonstrating a significant protective effect of higher education levels across these cohorts. Heterogeneity across studies was low (T^2^ = 0.0394). Stratifying the population in global north and global south had little impact in our findings, as did stratifying for sex, showing that the impact of education is not restricted to a specific segment.

**Conclusion:**

Our findings add to the literature on the important role of educational attainment as a protective factor for cognitive decline and dementia. Our data suggests that this notion is applicable throughout different populations, and that education may be a suitable target for global intervention on dementia prevention.